# Targeting Autophagy by MPT0L145, a Highly Potent PIK3C3 Inhibitor, Provides Synergistic Interaction to Targeted or Chemotherapeutic Agents in Cancer Cells

**DOI:** 10.3390/cancers11091345

**Published:** 2019-09-11

**Authors:** Chun-Han Chen, Tsung-Han Hsieh, Yu-Chen Lin, Yun-Ru Liu, Jing-Ping Liou, Yun Yen

**Affiliations:** 1 Department of Pharmacology, School of Medicine, College of Medicine, Taipei Medical University, Taipei 110, Taiwan; 2 Cell Physiology and Molecular Image Research Center, Wan Fang Hospital, Taipei Medical University, Taipei 110, Taiwan; 3 Joint Biobank, Office of Human Research, Taipei Medical University, Taipei 110, Taiwan; 4 School of Pharmacy, College of Pharmacy, Taipei Medical University, Taipei 110, Taiwan; 5 The Ph.D. Program for Cancer Molecular Biology and drug Discovery, College of Medical Science and Technology, Taipei Medical University, Taipei 110, Taiwan; 6 TMU Research Center of Cancer Translational Medicine, Taipei Medical University Taipei 110, Taiwan

**Keywords:** autophagy, gefitinib, gemcitabine, PIK3C3, synergism

## Abstract

Anticancer therapies reportedly promote pro-survival autophagy in cancer cells that confers drug resistance, rationalizing the concept to combine autophagy inhibitors to increase their therapeutic potential. We previously identified that MPT0L145 is a PIK3C3/FGFR inhibitor that not only increases autophagosome formation due to fibroblast growth factor receptor (FGFR) inhibition but also perturbs autophagic flux via PIK3C3 inhibition in bladder cancer cells harboring FGFR activation. In this study, we hypothesized that combined-use of MPT0L145 with agents that induce pro-survival autophagy may provide synthetic lethality in cancer cells without FGFR activation. The results showed that MPT0L145 synergistically sensitizes anticancer effects of gefitinib and gemcitabine in non-small cell lung cancer A549 cells and pancreatic cancer PANC-1 cells, respectively. Mechanistically, drug combination increased incomplete autophagy due to impaired PIK3C3 function by MPT0L145 as evidenced by p62 accumulation and no additional apoptotic cell death was observed. Meanwhile, drug combination perturbed survival pathways and increased vacuolization and ROS production in cancer cells. In conclusion, the data suggest that halting pro-survival autophagy by targeting PIK3C3 with MPT0L145 significantly sensitizes cancer cells to targeted or chemotherapeutic agents, fostering rational combination strategies for cancer therapy in the future.

## 1. Introduction

Autophagy is a catabolic process by which cells digest their own cellular contents, including amino acids, fatty acid, nucleotides and injured organelles [1]. There are mainly three types of autophagy, which are called macroautophagy, microautophagy and chaperone-mediated autophagy (CMA) [2,3]. Macroautophagy (hereafter referred to as autophagy) involves the formation of double-membrane structures known as autophagosomes to engulf damaged organelles or unused proteins, which ultimately fuse with lysosomes to degrade its contents. Microautophagy, on the other hand, engulfs cytoplasmic substances directly into the lysosome by inward folding of the lysosomal membrane. During CMA, the targeted proteins containing the recognition site for hsc70, allowing it to bind to chaperon and form the CMA-substrate/chaperone complex. The complex then binds to lysosome-associated membrane protein type 2A (LAMP-2A) and targeted proteins were unfolded and translocated across lysosomal membrane for degradation.

Anticancer therapies reportedly induce pro survival autophagy in cancer cells that confers drug resistance, which rationalizes the concept to combine autophagy inhibitors with different anticancer drugs or radiation to increase their therapeutic potential [4,5]. Several clinical trials make use of chloroquine or hydroxychloroquine to interfere with the fusion of autophagosomes and lysosomes [6,7]. Overall, these studies have demonstrated effective doses for hydroxychloroquine with tolerable side effects and have shown some promising therapeutic benefit [8]. However, higher dosages of hydroxychloroquine up to 1200 mg/d were needed to reach partial response or stable disease. Meanwhile, irreversible visual loss due to retinal toxicity from higher doses of hydroxychloroquine was also reported [9,10]. Therefore, there is still an unmet medical need to discover more potent and specific autophagy inhibitors.

PIK3C3 (alternatively termed VPS34), which belongs to class III PI3K, is an indispensable kinase during the process of autophagy [11]. Currently, two types of PIK3C3-containing complex were reported to be crucial to the progress of autophagy [12,13]. Both of them contain the core machinery of PIK3C3, PIK3R4 and BECN1. Complex I contains ATG14 and NRBF2, generating phosphatidylinositol 3-phosphate (PtdIns3P) to recruit the double-FYVE containing protein1 (DFCP-1) and the WD-repeat domain phosphoinositide-interacting proteins (WIPI) family proteins to the phagophore during autophagy initiation. Complex II, which contains UVRAG, is principally involved in autophagosome maturation and endosome maturation. Recently, autophagy has emerged as an attractive target for cancer therapy and selective PIK3C3 inhibitors were also developed as a new approach to block autophagy, such as the pyrimidinone (SAR405) and the bisaminopyrimidine (VPS34-IN1, PIK-III) compounds [14,15].

Previously, we reported that MPT0L145 is a first-in-class PIK3C3/FGFR inhibitor that not only increases autophagosome formation due to FGFR inhibition but simultaneously perturbs autophagy flux via PIK3C3 inhibition in bladder cancer cells harboring aberrant fibroblast growth factor receptor (FGFR) activation [16,17]. Dual inhibition of PIK3C3 and FGFR results in cytoplasmic vacuolization at peri-nuclear region, which promotes mitochondrial damage, reactive oxygen species (ROS) production and DNA damage. Importantly, the study revealed that MPT0L145 sensitizes the anticancer activities of several autophagy inducers, such as cisplatin, rapamycin and BGJ398 in bladder cancer cells. Therefore, we hypothesized that combined-use of MPT0L145 with agents that induce pro-survival autophagy may provide synthetic lethality in cancer cells without FGFR activation. The results showed that MPT0L145 synergistically sensitizes the anticancer effects of gefitinib and gemcitabine in non-small cell lung cancer A549 cells and pancreatic cancer PANC-1 cells, respectively. Mechanistically, drug combination increased incomplete autophagy due to impaired PIK3C3 function by MPT0L145 and no pronounced apoptotic cell death was observed. Additionally, drug combination perturbed survival pathways and increased ROS production in cancer cells. In conclusion, this study provides a rational combination strategy by targeting PIK3C3 to overcome drug resistance associated with pro-survival autophagy for cancer therapy.

## 2. Results

### 2.1. Combined-Use of MPT0L145 with Targeted or Chemotherapeutic Agents Provides Synthetic Lethality in Cancer Cells

**Figure 1:**
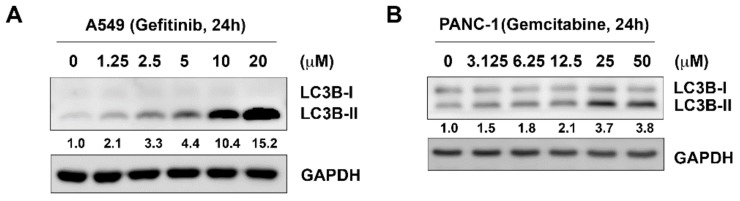
Effects of targeted or chemotherapeutic agents on autophagy in cancer cells. (**A**) A549 and (**B**) PANC-1 cells were treated with indicated drugs for 24h and subjected to western blot analysis by antibodies against LC3B and GAPDH.

**Figure 2:**
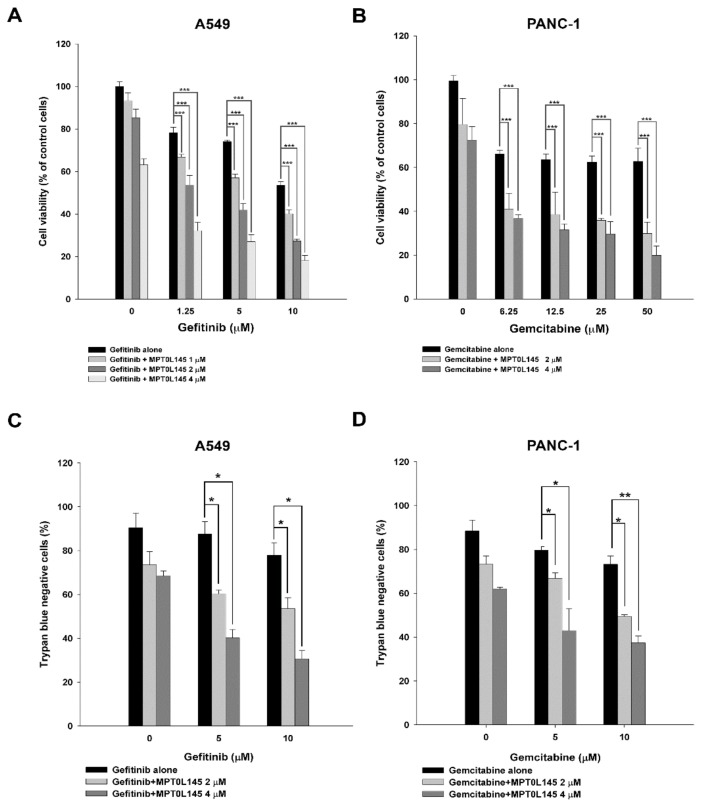
MPT0L145 sensitized cancer cells to targeted or chemotherapeutic agents. (**A**,**C**) A549 cells were treated with indicated concentrations of gefitinib in the absence or presence of MPT0L145 for 72h and subjected to MTT assay (**A**) or trypan blue exclusion assay (**C**). Data are expressed as means ± S.D. (*N* = 3, * *p* < 0.05, *** *p* < 0.001 compared to gefitinib alone). (**B**,**D**) PANC-1 cells were treated with indicated concentrations of gemcitabine in the absence or presence of MPT0L145 for 72h and subjected to MTT assay (**B**) or trypan blue exclusion assay (**D**). Data are expressed as means ± S.D. (*N* = 3, * *p* < 0.05, ** *p* < 0.01, *** *p* < 0.001 compared to gemcitabine alone).

Gefitinib is a first-line targeted therapy for non-small cell lung cancer in patients harboring active mutation of EGFR [18]. Gemcitabine has been successfully used as a chemotherapeutic agent in various malignancies, including pancreatic cancer [19]. However, both of them have been reported to induce pro-survival autophagy that confers drug resistance [4,5]. Elevated basal autophagy in gefitinib- or gemcitabine-resistant cells was also reported [20,21]. In this study, we chose A549 and PANC-1 cells to conduct the following experiments as they are primarily resistant to gefitinib and gemcitabine, respectively [22,23]. We first confirmed the effects of gefitinib and gemcitabine on autophagy in A549 and PANC-1 cells. The results showed that both gefitinib and gemcitabine were able to induce autophagy, as evident by the increased expression of LC3B-II (Figure 1A and 1B). These data confirmed that targeted- or chemo-therapeutic agents induce autophagy in cancer cells, which led us to hypothesize that targeting autophagy by a novel PIK3C3 inhibitor, MPT0L145 [16], might increase their sensitivity. The data revealed that neither gefitinib nor MPT0L145 decreased more than 50% of cell viability as a single agent in A549 cells. However, MPT0L145 synergistically sensitized the cells to gefitinib in a concentration-dependent manner and the combination index (CI) values are all below 1 (Figure 2A, Table S1). The same phenomenon was observed in PANC-1 cells by the combination of gemcitabine and MPT0L145 (Figure 2B, Table S1). We further confirmed the results by trypan blue exclusion assay, suggesting synergistic interaction of MPT0L145 with gefitinib or gemcitabine in A549 and PANC-1 cells, respectively (Figure 2C,D, Table S1). These results indicated that halting autophagy by MPT0L145 is a promising approach to increase the efficacy of targeted or chemotherapeutic agents in cancer cells.

### 2.2. PIK3C3 Knockdown Mimics the Effects of MPT0L145

**Figure 3:**
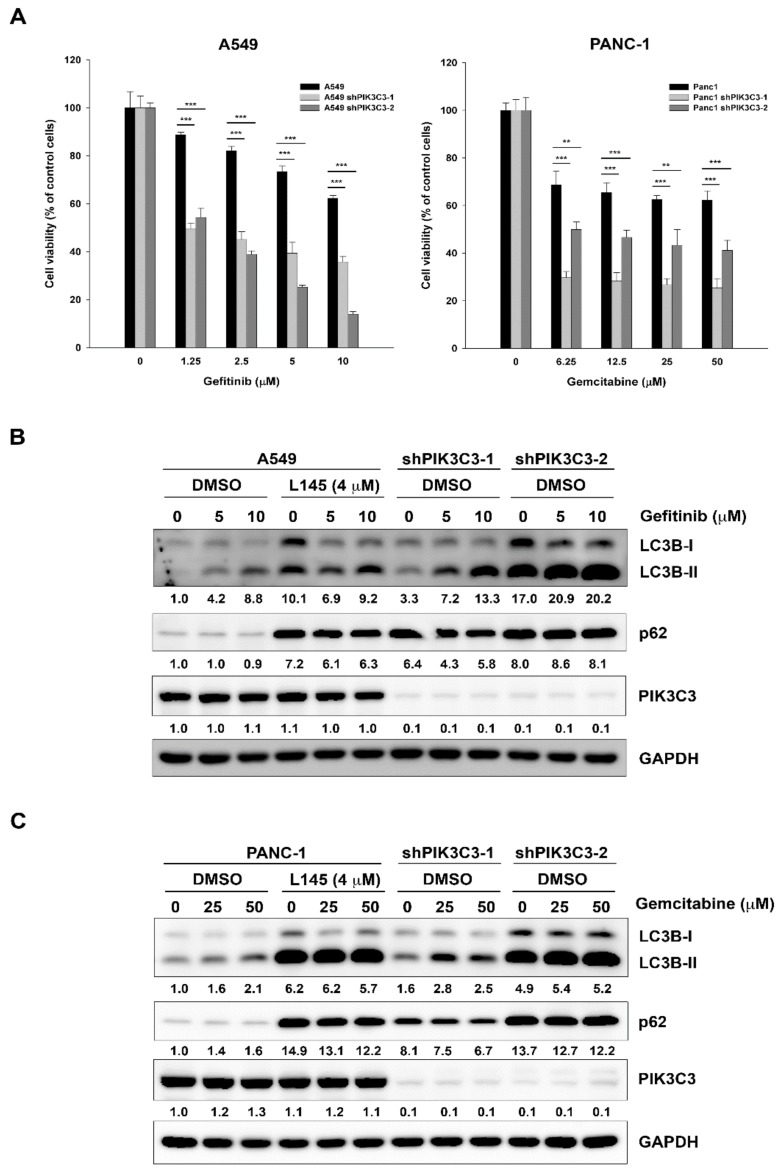
Effects of drug combination or PIK3C3-knockdown on autophagy in cancer cells. (**A**) PIK3C3 was stably knocked down in A549 (*left panel*) and PANC-1 cells (*right panel*) and exposed to indicated concentrations of gefitinib and gemcitabine, respectively. Cell viability of parental or PIK3C3-knockdown cells were measured by MTT assay. Data are expressed as means ± S.D. (*N* = 3, ** *p* < 0.01, *** *p* < 0.001 compared to wild-type group). (**B**) A549 cells were treated with gefitinib in the presence of MPT0L145 in parental cells or gefitinib alone in PIK3C3-knockdown cells for 24h and subjected to western blot analysis. (**C**) PANC-1 cells were treated with gemcitabine in the presence of MPT0L145 in parental cells or gemcitabine alone in PIK3C3-knockdown cells for 24h and subjected to western blot analysis.

To further confirm that the synergistic effects result from inhibition of PIK3C3, we stably knocked down PIK3C3 in A549 and PANC-1 cells via lentiviral transduction of shRNA targeting *PIK3C3* gene. The system displayed high knockdown efficiency between 80% to 90% in A549 (Figure S1A) and PANC-1 (Figure S1B) cells, with no appreciable effects on the growth rate. As shown in Figure 3A, knocking down of PIK3C3 increased the cytotoxic effects of gefitinib and gemcitabine in A549 and PANC-1 cells, respectively. To further examine the effects of drug combination on autophagy, we monitored the expression of LC3B-II and p62 by western blot analysis. In A549 cells, gefitinib increased the expression of LC3B-II in a concentration-dependent fashion (Figure 3B, lane 1–3). When combining with MPT0L145, autophagic flux was blocked as evident by the accumulation of p62 (Figure 3B, lane 4–6). Knocking down of PIK3C3 mimicked the effects of MPT0L145 (Figure 3B, lane 7–12). The same phenomenon was observed in PANC-1 cells by the combination of gemcitabine and MPT0L145 (Figure 3C). Together, MPT0L145 sensitized cancer cells to targeted or chemotherapeutic agents via inhibition of PIK3C3, which perturbed the process of autophagy.

### 2.3. Drug Combination Displays no Effect on Cell Cycle and Apoptosis

**Figure 4:**
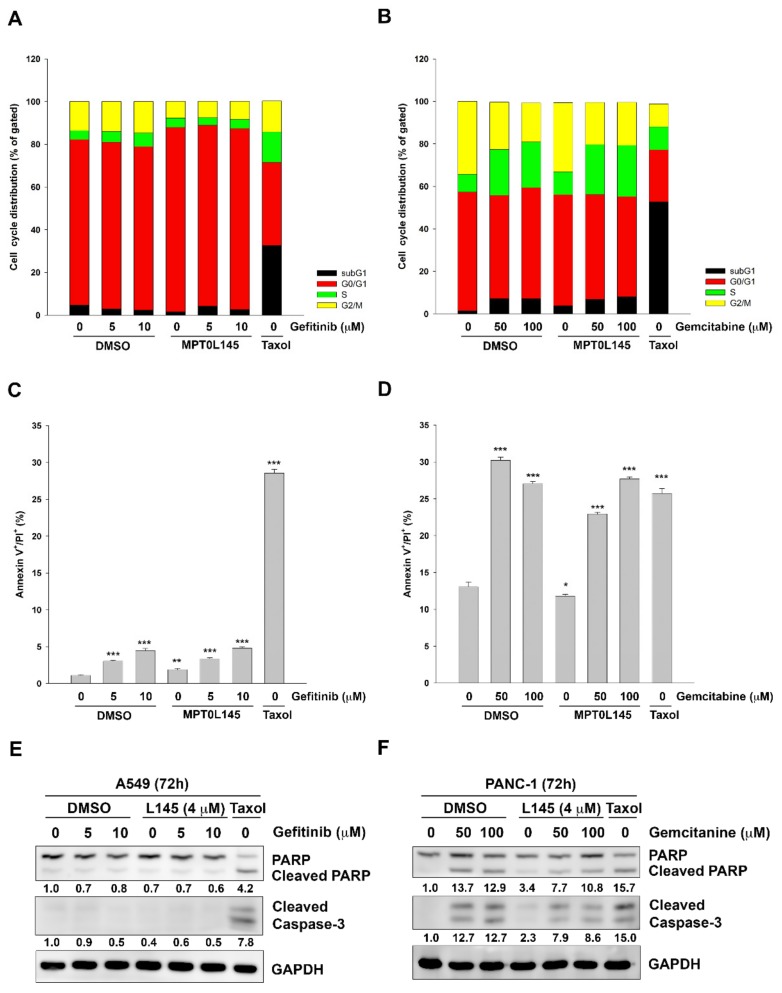
Effects of drug combination on cell cycle distribution and apoptosis. (**A**,**C**) A549 and (**B**,**D**) PANC-1 cells were respectively treated with MPT0L145 (4 μM) in the presence of gefitinib or gemcitabine for 72h. The cells were then stained with propidium iodide solution (**A**,**B**) or Annexin V-FITC/PI solution (**C**,**D**) and analyzed by flow cytometry. Paclitaxel (Taxol, 0.1 μM) was included as a positive control of apoptosis. (**E**) A549 and (**F**) PANC-1 cells were exposed to MPT0L145 (4 μM) in the presence or absence of gefitinib or gemcitabine, respectively for 72h. The cells were subjected to western blot analysis by using antibodies against PARP, caspase-3 and GAPDH.

To further examine the underlying mechanism of cell death induced by drug combination, we firstly analyzed the effects on cell cycle progression by PI staining and flow cytometry. In A549 cells, gefitinib alone slightly increased the cells in S phase. MPT0L145 alone slightly increased the cells in G_0_/G_1_ phase but the phenomenon was not further enhanced by the combination with gefitinib (Figure 4A). In PANC-1 cells, gemcitabine alone increased the cells in S and subG1 phase, accompanied by the decrease in G2/M phase. But the combination with MPT0L145 had no further effects on cell cycle distribution (Figure 4B). The data also revealed that apoptotic cell death was not further enhanced by combining with MPT0L145, as evidenced by Annexin V/PI staining method (Figure 4C and 4D). Moreover, the results were further confirmed in both A549 (Figure 4E) and PANC-1 (Figure 4F) cells by detecting the cleavage of PARP and caspase-3 where paclitaxel was included as a positive control. In summary, drug combination showed no further effects on cell cycle progression and apoptosis in cancer cells.

### 2.4. Drug Combination Perturbs Cell Survival Pathways in Cancer Cells

**Figure 5:**
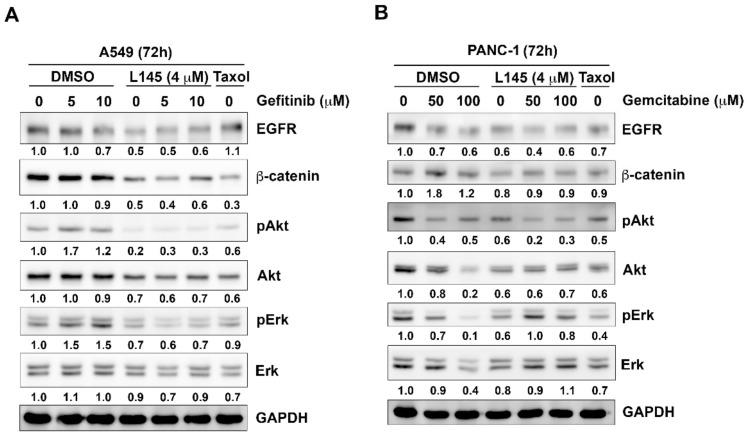
Effects of drug combination on cell survival pathways in cancer cells. (**A**) A549 and (**B**) PANC-1 cells were treated with MPT0L145 (L145, 4 μM) in combination with gefitinib or gemcitabine, respectively for 72h. The cells were then subjected to western blot analysis.

In non-small cell lung cancers, the signaling from EGFR to the phosphorylation of Akt and ERK was correlated with the sensitivity of gefitinib [22]. Moreover, overexpression of β-catenin is reportedly correlated to gefitinib-resistance [24,25]. In pancreatic cancers, ERK1/2 activity confers the resistance to gemcitabine [26]. Inhibition of AKT2 activity reportedly increases the sensitivity of gemcitabine in human pancreatic ductal adenocarcinoma [27]. Hence, we further examine the effects of drug combination on the protein levels of key molecules in cell survival pathways. The results revealed that gefitinib alone slightly decreases protein levels of EGFR and β-catenin, whereas pAKT and pErk were increased in A549 cells. However, protein expression of EGFR, β-catenin, pAKT and pERK and were decreased upon the treatment of gefitinib and MPT0L145 in A549 cells (Figure 5A). In PANC-1 cell, gemcitabine itself slightly decreased protein levels of EGFR and pAKT. The combination of gemcitabine and MPT0L145 further decreased the expression of EGFR and pAKT (Figure 5B). The effects on EGFR/pERK signaling pathway were also observed at lower concentrations of gefitinib or gemcitabine when combining with MPT0L145 (Figure S2A,B). Together, drug combination perturbs survival pathways in cancer cells, such as EGFR and β-catenin.

### 2.5. Drug Combination Increases Intracellular Vacuolization and Reactive Oxygen Species (ROS) Level

**Figure 6:**
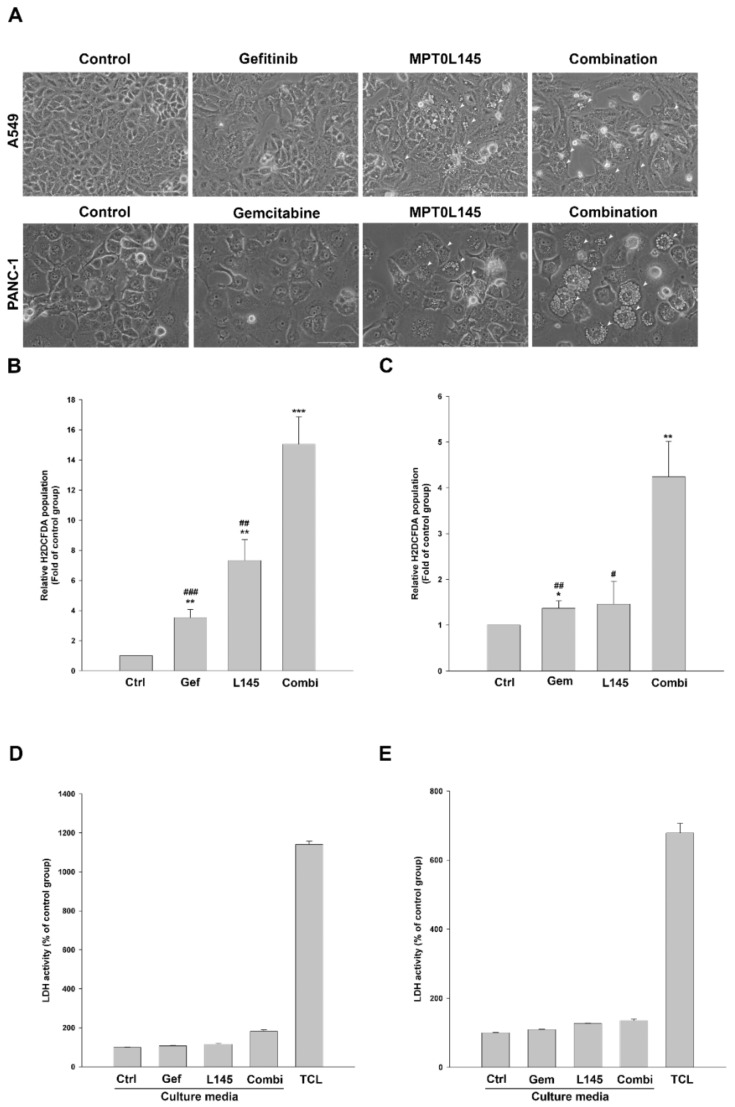
Drug combination increases intracellular vacuolization and reactive oxygen species (ROS) level in cancer cells. (**A**) A549 (upper panel) and PANC-1 (lower panel) cells were exposed to MPT0L145 (4 μM) alone and in combination with gefitinib (10 μM) or gemcitabine (100 μM) for 48h, respectively. Cell morphology was examined with the EVOS XL Core Cell Imaging System (Thermo Scientific). Intracellular vacuoles are indicated by white arrowheads. Scale bars: 100 μm. (**B**) A549 and (**C**) PANC-1 cells were treated with MPT0L145 (L145, 4 μM) in the presence of gefitinib (Gef, 10 μM) or gemcitabine (Gem, 100 μM), respectively for 72h. The cells were stained with H_2_DCFDA and intracellular ROS levels were measured by flow cytometry. Data are expressed as means ± S.D. (*N* = 3, * *p* < 0.05, ** *p* < 0.01 compared to control group; ^#^ *p* < 0.05, ^##^ *p* < 0.01, ^###^ *p* < 0.001 compared to combination group). (**D**) A549 and (**E**) PANC-1 cells were treated with MPT0L145 (L145, 4 μM) in the presence of gefitinib (Gef, 10 μM) or gemcitabine (Gem, 100 μM), respectively for 72h. Culture media or total cell lysates (TCL) from untreated cells, which served as maximum LDH release control, were collected and subjected to LDH analysis. Data were represented by the percentage of control group.

MPT0L145 reportedly increases cytoplasmic vacuolization at the peri-nuclear region, which partly contributes to cell death via ROS accumulation and DNA damage [16]. Therefore, we examine the effects of drug combination on morphological changes via phase-contrast microscopy as well as ROS production by staining with a fluorescent probe H_2_DCFDA. In A549 cells, MPT0L145 induced large phase-lucent vacuoles but the phenomenon was not further increased by the combination of gefitinib (Fig 6A, upper panel). MPT0L145 promoted the formation of small vacuoles in PANC-1 cells, which became larger when combined with gemcitabine (Figure 6A, lower panel). Meanwhile, the data suggested that the combination of gefitinib and MPT0L145 increased greater intracellular ROS level than each drug alone in A549 cells (Figure 6B). The same result was also observed in PANC-1 cells under the combination of gemcitabine and MPT0L145 (Figure 6C). ROS is reportedly one of the driving force to trigger necroptosis, a type of programmed necrosis [28]. However, the possibility of necrosis was excluded by detecting the release of LDH in culture media (Figure 6D,E). Together, we conclude that drug combination increased intracellular vacuolization and ROS accumulation in cancer cells.

## 3. Discussion

Targeted therapies or chemotherapeutic agents reportedly increase pro-survival autophagy in different types of cancer cells that results in drug resistance [4,5]. In several clinical trials, high doses of hydroxychloroquine were utilized to block autophagy by alkalinizing lysosomes that increased the therapeutic potential of anticancer drugs [6]. Overall, these studies have demonstrated effective doses for hydroxychloroquine with tolerable side effects and have shown some promising therapeutic benefit. However, there are some limitations of hydroxychloroquine that may be obstacles to successful clinical use. First, higher doses of hydroxychloroquine up to 1200 mg/d were used that only provide a partial response or stable disease [29]. However, irreversible visual loss due to retinal toxicity from high doses of hydroxychloroquine was reported [9,10]. Second, PPT1 was recently identified as a lysosomal target of hydroxychloroquine [30]. As the inactive mutations in *PPT1* gene lead to fatal neurodegeneration in infantile neuronal cerebral lipofuscinosis (INCL) patients, the second generation of chloroquine derivatives that penetrate the blood-brain barrier are more likely to have CNS toxicity in the future [31]. Therefore, there is still an unmet medical need for discovering potent and specific autophagy inhibitors.

Recently, specific inhibitors that perturb the autophagy-inducing proteins and lipid kinases were developed by targeting ULK1 and PIK3C3 (VPS34), respectively [14,15]. MPT0L145 is a highly potent inhibitor of PIK3C3 with a Kd value of 0.53 nM with minor activity to FGFR that interferes autophagy flux in FGFR-activating bladder cancer cells [16]. Base on the hypothesis, we aim to examine the effects of combined-use of MPT0L145 with anticancer drugs reportedly induce pro-survival autophagy to increase their therapeutic efficacy. The results showed that MPT0L145 synergistically sensitizes the anticancer effects of gefitinib and gemcitabine in A549 cells and PANC-1 cells, respectively. Knocking down of PIK3C3 mimicked the effects of MPT0L145 (Figure 2C), excluding the involvement of its FGFR inhibitory activity. It has been reported that chloroquine is able to overcome innate or acquired resistance to gefitinib and erlotinib in non-small-cell lung cancer cells [20,32,33]. Meanwhile, an autophagy inhibitor verteporfin reportedly enhances the antitumor effect of gemcitabine in a pancreatic ductal adenocarcinoma model *in vitro* and *in vivo* [34]. We also observed that other PIK3C3 inhibitors, such as 3-methyladenine (3-MA) and SAR-405, were able to provide synergisms with gefitinib in A549 cells and gemcitabine in PANC-1 cells (Figure S3,4). These findings support our findings that combined use of MPT0L145 with gefitinib or gemcitabine provides synergistic effects in lung and pancreatic cells. Therefore, this study proofed our concept that targeting PIK3C3 to overcome drug resistance associated with pro-survival autophagy for cancer therapy. Of note, higher concentrations of gefitinib and gemcitabine were used in the current study. Co-delivery of gefitinib and chloroquine by nanoparticles showed higher inhibition rates and enhance apoptosis than free drugs to overcome acquired resistance [35]. Therefore, it is important to solve the pharmacokinetic issue to improve the translational potential in the clinical setting.

Although combined use of autophagy inhibitors and anticancer agents has shown promising outcome in the clinical setting [8], the detailed mechanism of cancer cell death remains elusive. Liu et al. revealed that inhibition of autophagy causes elevated proteasomal activity leading to enhanced degradation of checkpoint kinase 1 (Chk1), which subsequently impaired the error-free DNA repair process, homologous recombination (HR). Therefore, the combination of autophagy inhibitors with agents that cause DNA damage may provide a strategy to induce synthetic lethality in cancer cells [36]. In human lung cancer cells, EGFR inhibition activates autophagy as a cytoprotective response [37]. Autophagy inhibition overcomes the resistance to EGFR inhibitors in lung cancer cells via Foxo3a activation or ER-stress, leading to apoptosis [38,39]. However, no additional apoptotic cell death was observed through the combination of MPT0L145 and gefitinib in A549 cells. Therefore, inhibition of autophagy flux by MPT0L145 may enhance the cytotoxicity of EGFR inhibitors via a unique type of cell death other than apoptosis. From our previous study, intracellular and mitochondrial ROS were elevated by the treatment of MPT0L145 and that associates with DNA damage response and cell death [16]. In the current study, we observed that intracellular vacuoles and ROS levels were increased under drug combination. As drug-induced vacuolization reportedly increases non-apoptotic cell death, resulting from the production of ROS [40,41]. JNK activation reportedly correlated with methuosis, a type of non-apoptotic cell death and mitochondrial ROS production [42,43]. These findings warrant further study on the underlying mechanisms of synergism by the combination of MPT0L145 with gefitinib or gemcitabine.

In the current study, we observed that MPT0L145 in combination with gefitinib or gemcitabine decreases the protein levels of EGFR and β-catenin (Figure 5A,B). It has been reported that EGFR is required for KRAS-induced pancreatic tumorigenesis and therefore served as a therapeutic target for pancreatic cancer [44,45]. Gemcitabine plus erlotinib showed benefit in advance pancreatic patients [46,47]. Moreover, wnt/β-catenin pathway confers chemoresistance in pancreatic cancers [48,49]. In line with our observations, MPT0L145 may provide benefit in sensitizing the clinical use of EGFR inhibitors or gemcitabine for the treatment of pancreatic cancer. Meanwhile, the mechanism by which the drug combination-mediated downregulation of EGFR and β-catenin is worthy to be elucidated in the future.

## 4. Materials and Methods

### 4.1. Cell Culture, Antibodies and Reagents

A549 cells were cultured in RPMI 1640 and Panc 1 cells were cultured in DMEM, supplemented with 10% (v/v) FBS and 1% (v/v) antibiotic-antimycotic solution (Thermo Fisher Scientific, Waltham, MA, USA) at 37 °C in a humidified incubator containing 5% CO_2_. MPT0L145 was synthesized by Dr. Jing-ping Liou according to previous publications [17] and is covered by PCT/US2016/043203 filed on 20 July 2016. Gefitinib, gemcitabine and paclitaxel were purchased from Cayman Chemical (Ann Arbor, MI, USA). 3-(4,5-Dimethylthiazol-2-yl)-2,5-diphenyltetrazolium bromide (MTT) was obtained from Sigma Chemical Corp (St. Louis, MO, USA). Antibodies against various proteins were obtained from the following sources: caspase-3, LC3B from Novus biologicals (Littleton, CO, USA); β-catenin, EGFR, PARP, pAKT, AKT, pErk and Erk from Cell Signaling Technology (Danvers, MA, USA) and PIK3C3 and GAPDH from Genetex (Irvine, CA, USA).

### 4.2. Cell Viability, Trypan Blue Exclusion Assay and Lactate Dehydrogenase (LDH) Assay

Cells were seeded in 96-well plates and exposed to indicated compounds for 72 h. Cell viability was examined by MTT assay as described previously [50]. For trypan blue exclusion assay, the cells were collected by trypsinization and diluted by 0.4% trypan blue solution (1:1). The percentage of trypan blue negative cells were determined by LUNA-II™ Automated Cell Counter (Logos Biosystems, Gyeonggi-do, Korea). For drug interaction study, the combination index (CI) value was generated from the fraction-affected value of each combination according to the Chou–Talalay method by the CompuSyn software (ComboSyn, Inc., Paramus, NJ, USA) [51]. A combination index value below 1 represents synergism. For LDH assay, cells were seeded in 96-well plates and treated with drugs at indicated concentrations for 72 h, followed by measuring LDH release in culture media by using the CytoTox 96 Non-Radioactive Cytotoxicity Assay Kit (Promega) according to the manufacturer’s protocol. 

### 4.3. Cell Cycle and Apoptosis Analysis

Cells were seeded in 6-well plates and treated with indicated compounds for 72 h. The cells were trypsinized, washed one time by PBS and fixed with ethanol (70%) at −20 °C overnight. The cells were pelleted by centrifugation and incubated in 0.1 mL of phosphate-citric acid buffer (0.2 M NaHPO4, 0.1 M citric acid and pH 7.8) for 15 min at room temperature and then re-suspended in propidium iodide staining buffer containing Triton X-100 (0.1%, v/v), RNase A (100 μg/mL) and propidium iodide (80 μg/mL) for 30 minutes in the dark. Cell cycle distribution was analyzed by flow cytometry with CellQuest software (Becton Dickinson, Mountain View, CA, USA) according to previous published methods [17]. For the detection of apoptosis, the cells were harvested by trypsinization and subjected to the staining of Annexin V-FITC/PI for 15 minutes according to the manufacturer’s instruction (Thermo Fisher Scientific, Waltham, MA, USA). The cells were analyzed by flow cytometry with CellQuest software. The cells that were positive for Annexin V and PI were considered late apoptotic cells.

### 4.4. Intracellular ROS Analysis

Cells were seeded in 6-well plates, exposed to indicated compounds for 72 h and harvested by trypsinization. Then the cells were stained with 0.1 μM H_2_DCFDA (Cat#10058; Biotium, Fremont, CA, USA) at 37 °C for 20 minutes. After washing with PBS three times, cells were subjected to ROS detection via flow cytometry with CellQuest software according to the manufacturer’s instructions (Becton Dickinson, Mountain View, CA, USA).

### 4.5. Western Blot Analysis and Lentiviral Expression System

The cells were seeded in 6-well plates or 60 mm dishes and exposed to different compounds for indicated times. After treatment, equal amounts of protein were separated via SDS–PAGE, transferred to PVDF membrane and immunoblotted with specific antibodies, as described previously [16]. Band intensities were measured by the software image J [52]. Band intensities were normalized by internal control and the data was represented as fold of control group. The whole blots were shown in Supplementary Figures S5 and S6. Lentiviral particles containing shRNA plasmids of shPIK3C3 (TRCN0000296101, TRCN0000310259) were purchased from the National RNAi Core Facility (Academia Sinica, Taiwan). Stable cell lines were selected by the treatment of puromycin (InvivoGen; San Diego, CA, USA).

### 4.6. Statistical Analysis

Each experiment was performed independently with at least two biological replicates. Data in the bar graphs are presented as means ± S.D. and analyzed by using the Student’s t-Test. *p* values less than 0.05 were considered significant.

## 5. Conclusions

In this study, we present the results showing that MPT0L145 synergistically sensitizes the anticancer effects of gefitinib and gemcitabine in non-small cell lung cancer A549 cells and pancreatic cancer PANC-1 cells, respectively. Mechanistically, drug combination increased incomplete autophagy due to impaired PIK3C3 function by MPT0L145. There was no additional apoptosis but perturbed survival pathways and increased ROS production were observed after combining with MPT0L145. We conclude that halting pro-survival autophagy by targeting PIK3C3 with MPT0L145 significantly sensitizes cancer cells to targeted or chemotherapeutic agents, fostering rational combination strategies for cancer therapy in the future.

## Supplementary Materials

The following are available online at https://www.mdpi.com/2072-6694/11/9/1345/s1, Figure S1: Knockdown efficiency of shRNAs against PIK3C3 and their effects on cell proliferation, Figure S2: Effects of drug combination on cell survival pathways in cancer cells, Figure S3: Known PIK3C3 inhibitors sensitized A549 cells to gefitinib, Figure S4: Known PIK3C3 inhibitors sensitized PANC-1 cells to gemcitabine, Table S1: The combination index (CI) values of different drug combinations in A549 and PANC-1 cells.
