# Supplementary material for: Targeting Autophagy by MPT0L145, a Highly Potent PIK3C3 Inhibitor, Provides Synergistic Interaction to Targeted or Chemotherapeutic Agents in Cancer Cells
Source: Cancers (Basel). 2019 Sep 11;11(9):1345. doi: 10.3390/cancers11091345 (PMC6770340; doi:10.3390/cancers11091345)
Supplement: Supplementary file 1 [file cancers-11-01345-s001.pdf]

cancers

MDPI

Supplementary materials: Targeting Autophagy by MPT0L145, a Highly Potent PIK3C3 Inhibitor, Provides Synergistic Interaction to Targeted or Chemotherapeutic Agents in Cancer Cells

Table S1. The combination index (CI) values of different drug combinations in A549 and PANC-1 cells.

**A549 (72h, MTT assay)**				**PANC-1 (72h, MTT assay)**			
**Gefitinib (μM)**	**MPT0L145 (μM)**	**Effect**	**CI**	**Gemcitabine (μM)**	**MPT0L145 (μM)**	Effect	CI
1.25	1.0	0.333	0.511	6.25	2.0	0.590	0.0492
5.00	1.0	0.430	0.625	12.5	2.0	0.641	0.0338
10.0	1.0	0.599	0.340	25	2.0	0.642	0.0335
1.25	2.0	0.464	0.451	50	2.0	0.701	0.0210
5.00	2.0	0.581	0.394	6.25	4.0	0.632	0.0723
10.0	2.0	0.727	0.242	12.5	4.0	0.685	0.0479
1.25	4.0	0.678	0.426	25	4.0	0.703	0.0413
5.00	4.0	0.730	0.381	50	4.0	0.801	0.0163
10.0	4.0	0.819	0.271				
**A549 (72h, Trypan blue exclusion assay)**				**PANC-1 (72h, Trypan blue exclusion assay)**			
Gefitinib (μM)	MPT0L145 (μM)	Effect	CI	Gemcitabine (μM)	MPT0L145 (μM)	Effect	CI
5.00	2.0	0.397	0.408	25	2.0	0.332	0.936
10.0	2.0	0.465	0.417	50	2.0	0.506	0.381
5.00	4.0	0.598	0.137	25	4.0	0.571	0.391
10.0	4.0	0.694	0.138	50	4.0	0.626	0.308

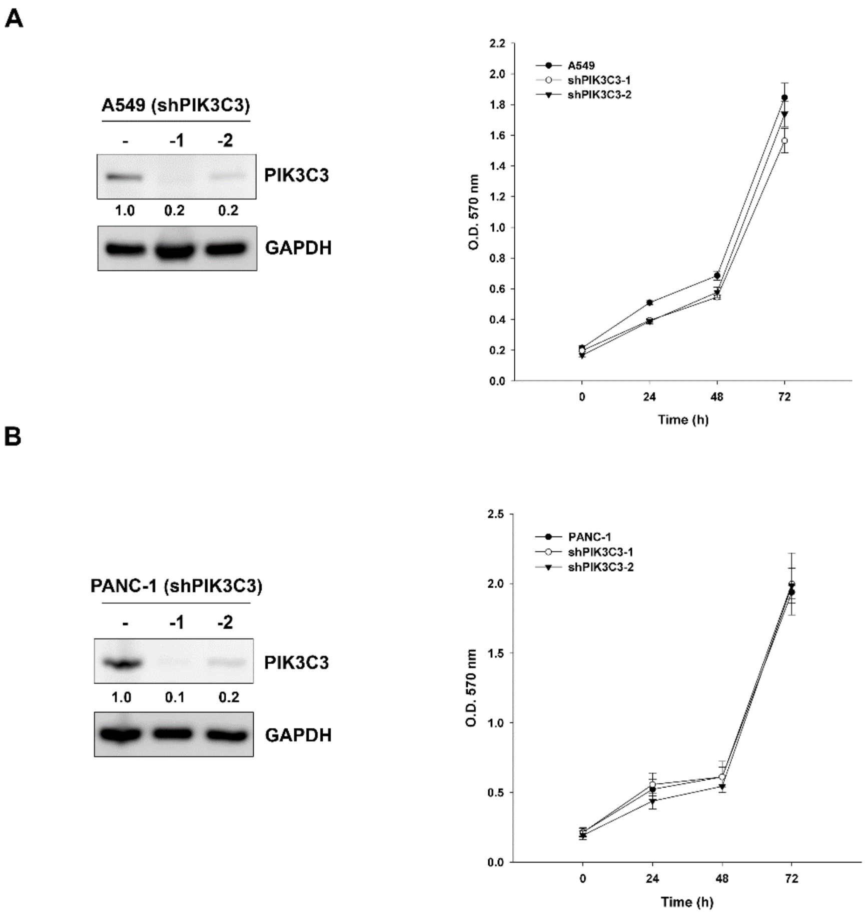

Figure S1. Knockdown efficiency of shRNAs against PIK3C3 and their effects on cell proliferation. (**A**) A549 and (**B**) PANC-1 cells were transduced with shPIK3C3-1 or shPIK3C3-2 by lentivirus. Stable cell lines were obtained by puromycin selection (2 μg/mL), and the cell lysates were subjected to western blot analysis (*left panel*). The effects of PIK3C3-knockdown on cell proliferation were analyzed by MTT assay at different time points (*right panel*).

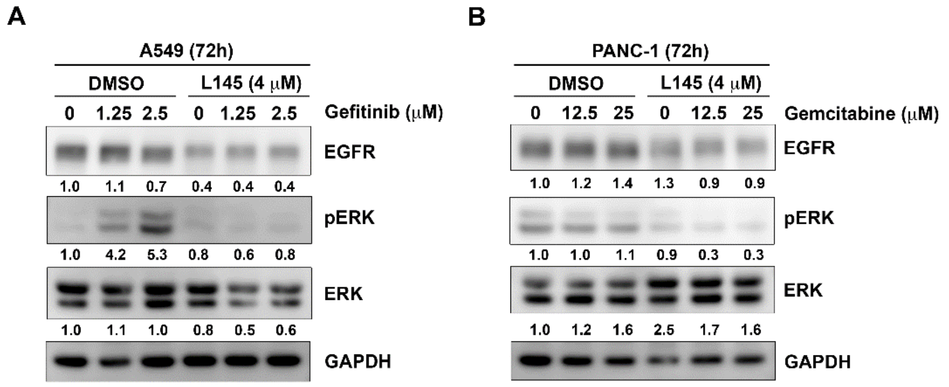

Figure S2. Effects of drug combination on cell survival pathways in cancer cells. (**A**) A549 and (**B**) PANC-1 cells were treated with MPT0L145 (L145, 4 μM) in combination with lower concentrations of gefitinib or gemcitabine, respectively for 72h. The cells were then subjected to western blot analysis.

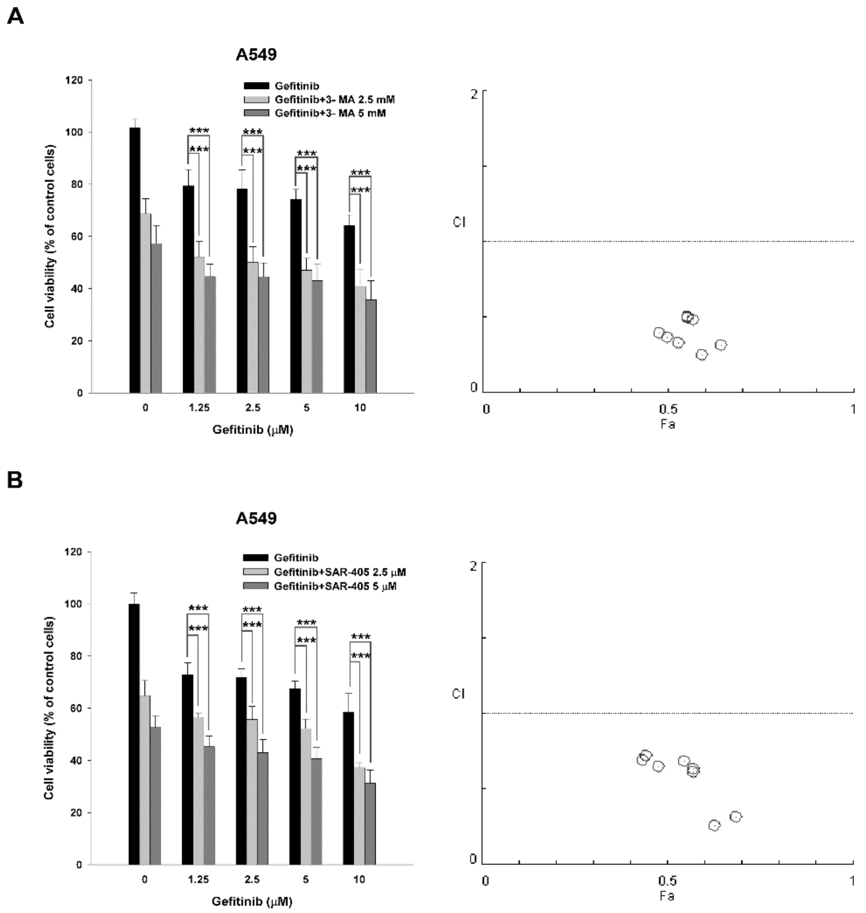

Figure S3. Known PIK3C3 inhibitors sensitized A549 cells to gefitinib. A549 cells were treated with indicated concentrations of gefitinib in the absence or presence of 3-methyladenine (3-MA) (**A**) or SAR405 (**B**) for 72h, and subjected to MTT assay. Data are expressed as means ± S.D. ($N = 3$, *** $p < 0.001$ compared to gefitinib alone). The combination index (CI) values were calculated by CompuSyn software (right panel).

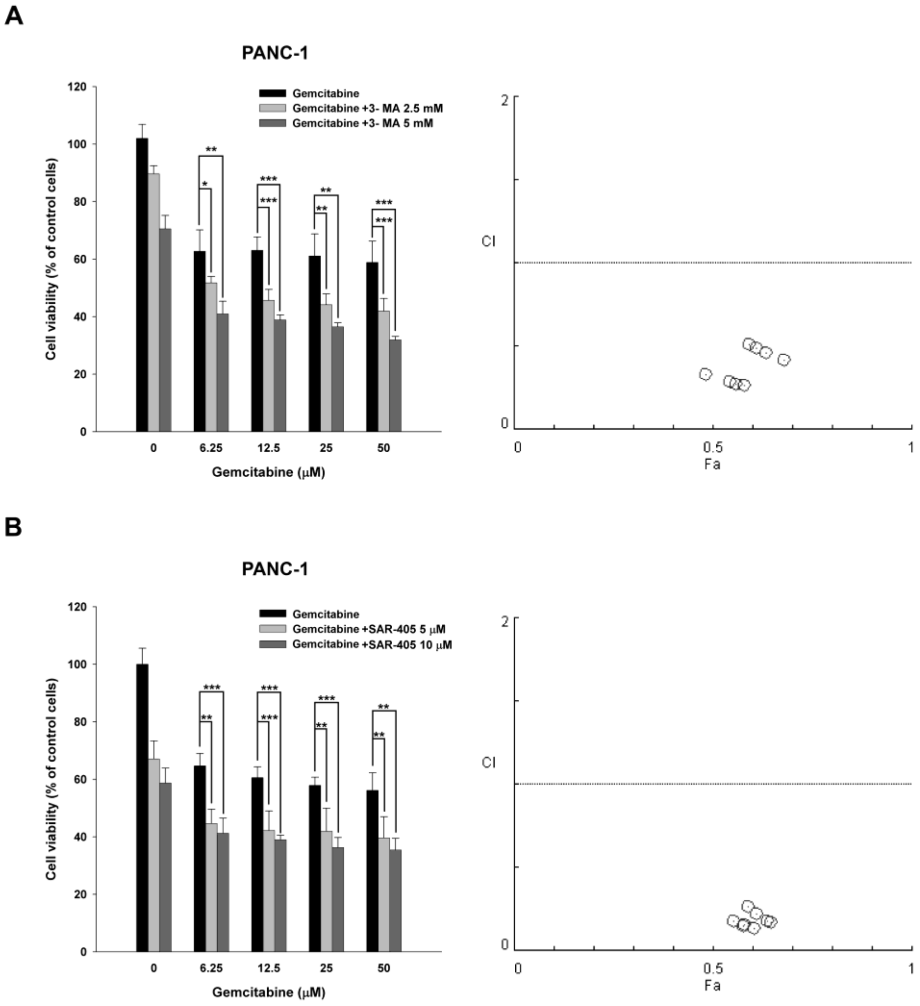

Figure S4. Known PIK3C3 inhibitors sensitized PANC-1 cells to gemcitabine. PANC-1 cells were treated with indicated concentrations of gemcitabine in the absence or presence of 3-methyladenine (3-MA) (**A**) or SAR405 (**B**) for 72h, and subjected to MTT assay. Data are expressed as means ± S.D. (N = 3, ** $p < 0.01$, *** $p < 0.001$ compared to gemcitabine alone). The combination index (CI) values were calculated by CompuSyn software (right panel).

Western blots:

Fig. 1A

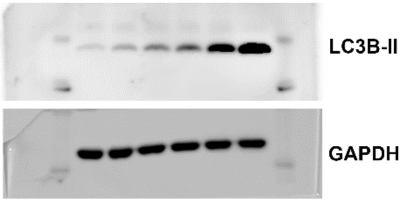

Fig. 1B

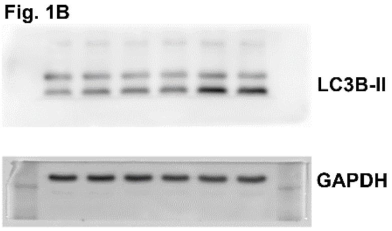

Fig. 3A

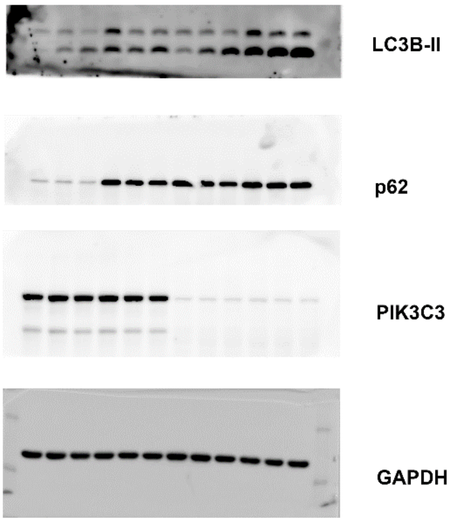

Fig. 3B

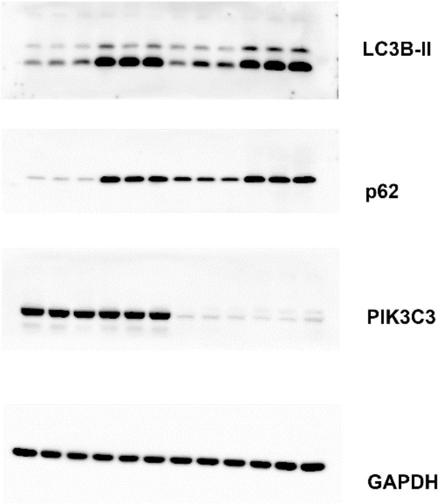

Fig. 4C

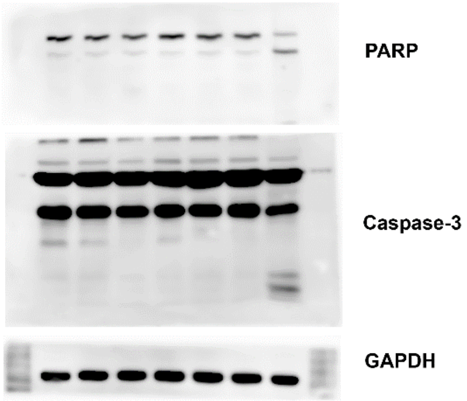

Fig. 4D

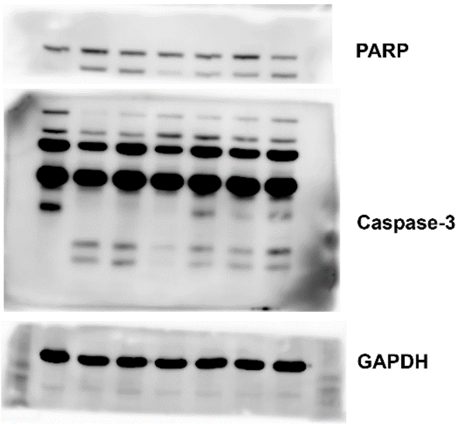

Fig. 5A

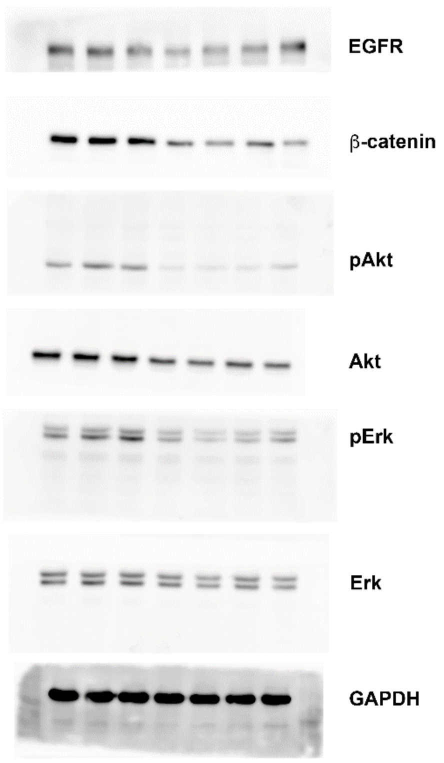
EGFR
β-catenin
pAkt
Akt
pErk
Erk
GAPDH

Fig. 5B

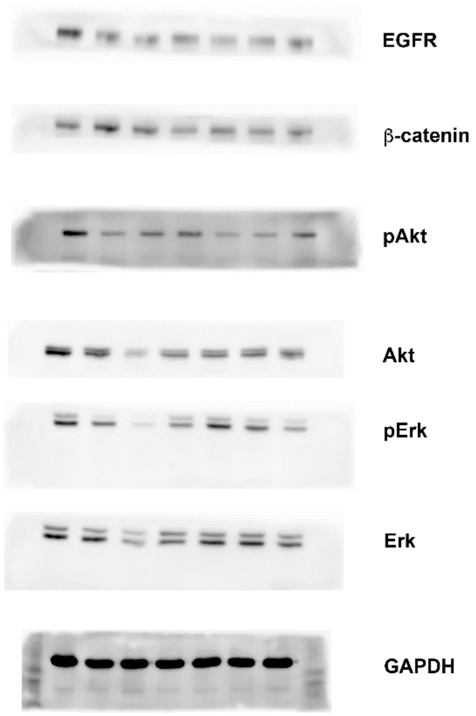
EGFR
β-catenin
pAkt
Akt
pErk
Erk
GAPDH
